# Intestinal flora study reveals the mechanism of Danggui Shaoyao San and its decomposed recipes to improve cognitive dysfunction in the rat model of Alzheimer’s disease

**DOI:** 10.3389/fcimb.2023.1323674

**Published:** 2023-11-21

**Authors:** Yijie Jin, Si Liang, Jiakang Qiu, Jing Jin, Yujia Zhou, Yaqi Huang, Chunxiang He, Wenjing Yu, Sisi Deng, Shaowu Cheng, Zhenyan Song

**Affiliations:** ^1^ School of Integrated Chinese and Western Medicine, Hunan University of Chinese Medicine, Changsha, Hunan, China; ^2^ Key Laboratory of Hunan Province for Integrated Traditional Chinese and Western Medicine on Prevention and Treatment of Cardio-Cerebral Diseases, Hunan University of Chinese Medicine, Changsha, Hunan, China

**Keywords:** Alzheimer’s disease, Danggui-Shaoyao-San, intestinal flora, cognitive dysfunction, traditional Chinese medicine

## Abstract

**Background:**

Alzheimer’s disease (AD), characterized by a severe decline in cognitive function, significantly impacts patients’ quality of life. Traditional Chinese Medicine (TCM) presents notable advantages in AD treatment, closely linked to its regulation of intestinal flora. Nevertheless, a comprehensive exploration of the precise role of intestinal flora in AD remains lacking.

**Methods:**

We induced an AD model through bilateral intracerebroventricular injection of streptozotocin in rats. We divided 36 rats randomly into 6 groups: sham-operated, model, Danggui Shaoyao San (DSS), and 3 DSS decomposed recipes groups. Cognitive abilities were assessed using water maze and open field experiments. Nissl staining examined hippocampal neuron integrity. Western blot analysis determined synaptoprotein expression. Additionally, 16S rDNA high-throughput sequencing analyzed intestinal flora composition.

**Results:**

DSS and its decomposed recipe groups demonstrated improved learning and memory in rats (*P*<0.01). The open field test indicated increased central zone residence time and locomotor activity distance in these groups (*P*<0.05). Furthermore, the DSS and decomposed recipe groups exhibited reduced hippocampal neuronal damage and increased expression levels of synapsin I (*P*<0.05) and PSD95 (*P*<0.01) proteins. Alpha and Beta diversity analyses showed that the intestinal flora species richness and diversity in the DSS and decomposed recipe groups were similar to those in the sham-operated group, signifying a significant restorative effect (*P*<0.05).

**Conclusion:**

The combination of DSS and its decomposed recipes can reduce the abundance of harmful gut microbiota, leading to improvements in cognitive and learning abilities.

## 1 Introduction

Alzheimer’s disease (AD) is the most common form of dementia in the elderly, characterized by a decline in cognitive abilities and independent learning and memory functions (Scheltens et al., 2021). Currently, the pathogenesis of AD remains unclear, and there are no effective drugs available to reverse its symptoms, which poses a pessimistic outlook for the development of clinical anti-AD medications (Alzheimer’s Association, 2022). It has been suggested that altering the abundance and diversity of gut microbiota can slow down the pathological progression of AD (Liu et al., 2023a). The connection between gut microbiota and AD has garnered considerable attention, as the gut-brain axis facilitates bidirectional communication between the gut and the brain through immune, endocrine, circulatory, and neural mechanisms (Doifode et al., 2021; Chen et al., 2022). Changes in gut microbiota composition, influenced by increased gut barrier permeability and activation of immune cells, can lead to compromised blood-brain barrier function, promoting neuroinflammation, neuronal loss, and neurodegeneration, ultimately contributing to AD (Megur et al., 2020). The emergence of the concept of the “gut-brain axis” has opened up new research directions for the development of AD treatments.

Traditional Chinese Medicine (TCM) exhibits a unique “multicomponent, multitarget” effect, leveraging its advantage in holistic treatment and holds promise as an effective approach for treating AD (Kim et al., 2023). Danggui Shaoyao San (DSS), a classic TCM prescription, is known for its liver-nourishing, blood-tonifying, and spleen-invigorating properties (Fu et al., 2016). Modern clinical and experimental studies have found that DSS can improve learning and memory functions in dementia patients through anti-inflammatory, anti-apoptotic, and antioxidant stress pathways (Lan et al., 2012; Fu et al., 2016; Huang et al., 2020; Song et al., 2020; Song et al., 2021a). Although numerous studies have demonstrated the significant therapeutic effects of DSS in improving cognitive impairments in AD, its mechanism of action remains unclear. The latest research indicates that the gut-brain axis has formed a bidirectional communication system between the gastrointestinal tract and cognitive brain regions (Liu et al., 2024). Traditional herbal medicine offers hope for the treatment of neurodegenerative diseases, especially AD, by correcting the imbalance in the gut microbiota (Li et al., 2023; Ma et al., 2023; Tang et al., 2023). TCM prescriptions, individual herbs, and compounds are involved in regulating the gut microbiota and its core metabolites(Hu and Wu, 2023; Liang et al., 2023; Liu et al., 2023b; Ma et al., 2023; Su et al., 2023). They further influence various pathways, including the hypothalamus-pituitary-adrenal axis, inflammation, and immune processes, thereby improving cognitive dysfunction in Alzheimer’s disease (Sun et al., 2022; Zhan et al., 2023).

Study on compatibility of TCM prescriptions, as one of the primary methods in the modern experimental research of TCM prescriptions, effectively elucidate the scientific rationale behind the structural compatibility of compound prescriptions (Zhu et al., 2021; Li et al., 2024). From a chemical or pharmacological perspective, the composition of a decoction becomes relatively complex after compatibility, and the active ingredients undergo changes during processes such as decoction, circulation, and metabolism. This study takes the premise of “treating diseases according to their pathogenesis, and decocting formulas according to established principles” (Traditional Chinese medicine theory) and divides the 6 herbs in DSS into different groups based on their respective functions (**Table 1**): blood-nourishing and blood-activating group (Danggui, Shaoyao, Chuanxiong), spleen-invigorating and dampness-eliminating group (Fuling, Zexie, Baizhu), and blood-nourishing and spleen-invigorating group (Shaoyao, Baizhu). The study aims to explore the improvements in cognitive function and the regulatory effects on gut microbiota exerted by both the DSS whole prescription and its decomposed recipes. Simultaneously, it provides experimental evidence for the rationality of the compatibility of DSS in the treatment of AD and offers new perspectives for clinical prescription formulation and medication guidance.

**Table 1:** Component herbs of DSS.

Chinese name	Latin name	Family	Part used	Ingredient ratio
Danggui	*Angelica sinensis (Oliv.)* Diels	Umbelliferae	Radix	3
Shaoyao	*Paeonia lactiflora* Pall	Buttercup	Radix	16
Fulin	*Poria cocos (Schw.)* Wolf	Polyporaceae	Sclerotium	4
Baizhu	*Atractylodes macrocephala* Koidz	Compositae	rhizome	4
Zexie	*Alisma orientalis (Sam.)* Juzep	Alismataceae	stem tuber	8
Chuanxiong	*Ligusticum chuanxiong* Hort	Umbelliferae	rhizome	8

## 2 Materials and methods

### 2.1 Animals

Male SD rats (36 in total) weighing between 150 and 200 g were purchased from Hunan Sleich Jingda Experimental Animal Co., Ltd. The animals were provided with a certificate of compliance for experimental animal production (SCXK [Xiang] 2021-0002) and housed in the Animal Experimental Center of Hunan University of Chinese Medicine. The experimental protocol was approved by the Ethics Committee of Hunan University of Chinese Medicine, with an ethics approval number of LLBH-202103180001. The rats had free access to water and were maintained under constant temperature conditions with a 12-hour light-dark cycle.

### 2.2 Drugs and reagents

DSS herbal ingredients, including Shaoyao, Chuanxiong, Zexie, Fuling, Danggui, and Baizhu, as well as the decoction ingredients, were purchased from the Chinese Medicine Pharmacy of the First Affiliated Hospital of Hunan University of Chinese Medicine, with batch number 19615. Streptozotocin (STZ) was obtained from Beijing Soleibao Technology Co., Ltd., with batch number S8050.

### 2.3 Preparation and identification of medicinal substances

Danggui Shaoyao San was prepared using a water extraction method according to a previously established protocol in our research group (Song et al., 2020). The freeze-dried powders for each decoction group were prepared in proportion to the original formula using the same method.

### 2.4 Animal modeling

Streptozotocin (STZ) was dissolved in citric acid buffer to prepare an STZ injection solution with a dosage of 2.4 mg/kg. Fasting for 12 hours, the modeling was initiated by anesthetizing the rats with 10% chloral hydrate. Using a stereotaxic instrument and a microsyringe, the lateral ventricles of the rats were located according to the stereotaxic ATLAS of rat brain (6th edition) and injected with STZ within 5 minutes on each side at a constant speed of 5 μL. The sham-operated group received an equivalent amount of saline injection. After the injection, the needle was kept in place to prevent leakage of STZ. The incision was sutured after the needle was removed, and the rats were returned to their cages for recovery. The Morris water maze test was performed on the 6th day to evaluate the success of the modeling (Song et al., 2021a).

### 2.5 Animal grouping and drug intervention

In this study, the Danggui Shaoyao San group and three 3 DSS decomposed recipes groups were set up, and the 3 DSS decomposed recipes groups were set as follows: (Danggui, Shaoyao, Chuanxiong: which called as DSC in this study) have shown blood-nourishing and blood-activating, (Fuling, Baizhu, Zexie: which called as FBZ in this study) have shown spleen-invigorating and dampness-eliminating, and (Shaoyao, Baizhu: which called as SB in this study) have shown blood-nourishing and spleen-invigorating (Fu et al., 2016; Cui and Zhao, 2021).

The 36 rats were randomly divided into 6 groups: sham-operated group (Sham), STZ injected group (AD model), STZ-injected DSS group (AD-DSS), and 3 DSS decomposed recipes groups: STZ-injected DSC group (AD-DSC), and STZ-injected FBZ group (AD-FBZ), STZ-injected SB group (AD-SB). The oral administration of drugs began on the third day after modeling and lasted for 14 days. The model group received an equivalent amount of saline by oral gavage. The dosages for the Danggui Shaoyao San group and the decoction groups were calculated based on the original formula of Danggui Shaoyao San (24 g·kg^-1^·d^-1^) and adjusted according to the body surface area and previous preliminary studies (Yang et al., 2023).

### 2.6 Morris water maze test

The water maze was divided into four quadrants, each labeled accordingly. An escape platform was fixed 1 cm beneath the water surface in the first quadrant. The rats were placed into the water maze from different starting positions and the time taken to climb onto the platform and stay there for 2 seconds was recorded. If a rat failed to reach the platform within 60 seconds, it was guided to the platform and allowed to stand for 10 seconds to assist with memory formation. The learning sessions were conducted twice a day for 5 consecutive days. On the 6th day, the platform was removed, and the rats were tested using the same method, with the time spent in the first quadrant and the number of crossings recorded. The trajectory of each rat was analyzed by Morris water maze video analysis system (Beijing Zhongshi Dichuang Technology Development Co., Ltd.).

### 2.7 Open field test

The open field test was conducted in a rectangular arena divided into three concentric squares. The innermost square represented the central zone, and a camera was fixed above to record the movement trajectory of the rats. Recording commenced once each rat was positioned at the center of the arena, and any excrement was promptly removed post-experiment to eliminate residual odors and prevent interference with subsequent rats. The trajectory of each rat was analyzed by Labmaze Animal Behavior Analysis Software V3.0 (Beijing Zhongshi Dichuang Technology Development Co., Ltd.).

### 2.8 Sample collection

Rats were anesthetized with 3% sodium pentobarbital, and their chest cavities were exposed. The heart was perfused with physiological saline at 4°C until the liver turned pale. Subsequently, the rats were decapitated, and their skulls were opened to access the brain. Brain tissue was placed on an aluminum foil-lined cold plate, and a surgical blade was used to separate the left and right hemispheres. The crescent-shaped hippocampal tissue was visible on the cut surface. Delicate ophthalmic forceps were employed to gently lift the crescent-shaped tissue from the right hemisphere. Once isolated, the hippocampal tissue was placed in cryovials and flash-frozen in liquid nitrogen before being stored in a -80°C freezer for future molecular biology studies. The left hemisphere brain tissue was fixed in a 4% paraformaldehyde solution for histological examination.

### 2.9 Nissl staining

The half-brain samples were fixed, embedded in paraffin, and sectioned. After deparaffinization and rehydration, the sections were stained with 1% cresyl violet at 37°C for 25 minutes. Differentiation was performed in 95% ethanol for 30 seconds, followed by gradient dehydration, neutral gum mounting, and coverslipping.

### 2.10 Western blot

Total protein was extracted from the hippocampal tissue using the RIPA method, and the protein concentration was determined using the BCA assay kit. Electrophoresis and PVDF membrane transfer were performed. The membrane was blocked with 5% skim milk, incubated overnight at 4°C with primary antibodies (1:1000), incubated with secondary antibodies at 37°C for 1 hour, and visualized using an imaging system with the ECL method. Image J software was used for image analysis.

### 2.11 DNA extraction and PCR amplification of intestinal content

Total DNA of the gut microbiota was extracted from the rat colon contents using the CTAB method, followed by quality control and quantification. PCR amplification targeting the V3-V4 variable region of the 16S rRNA gene was performed using primers 341F (5’-CCTACGGGNGGCWGCAG-3’) and 805R (5’-GACTACHVGGGTATCTAATCC-3’), with triplicate reactions for each sample (Logue et al., 2016).

### 2.12 Quantification and sequencing of PCR products

PCR products were recovered from a 2% agarose gel and quantified. The samples were subjected to paired-end sequencing (2×250 bp) using the NovaSeq 6000 sequencer, with the corresponding NovaSeq 6000 SP Reagent Kit.

### 2.13 Data processing

The obtained sequencing data were split according to the barcode information, and the adapter and barcode sequences were removed. Length filtering and denoising were performed using the DADA2 algorithm. ASV (feature) sequence and abundance tables were obtained. Alpha and beta diversity analyses of the gut microbiota were conducted based on the obtained feature sequences and abundance tables. Species annotation was performed using the SILVA database with the NT-16S database, and the abundance of each species in each sample was statistically analyzed based on the ASV abundance table. The confidence threshold for annotation was set at 0.7 (Wang et al., 2022).

### 2.14 Statistical analysis

Measurement data were expressed as mean ± standard deviation. One-way analysis of variance (ANOVA) was conducted, and statistical graphs were created using GraphPad 8.0.1. A significance level of *P*<0.05 was considered statistically significant.

## 3 Results

### 3.1 Impact of Danggui-Shaoyao-San on cognitive function

#### 3.1.1 Effects of Danggui Shaoyao San and its decomposed recipes on cognitive function in AD rats

**Figure 1:**
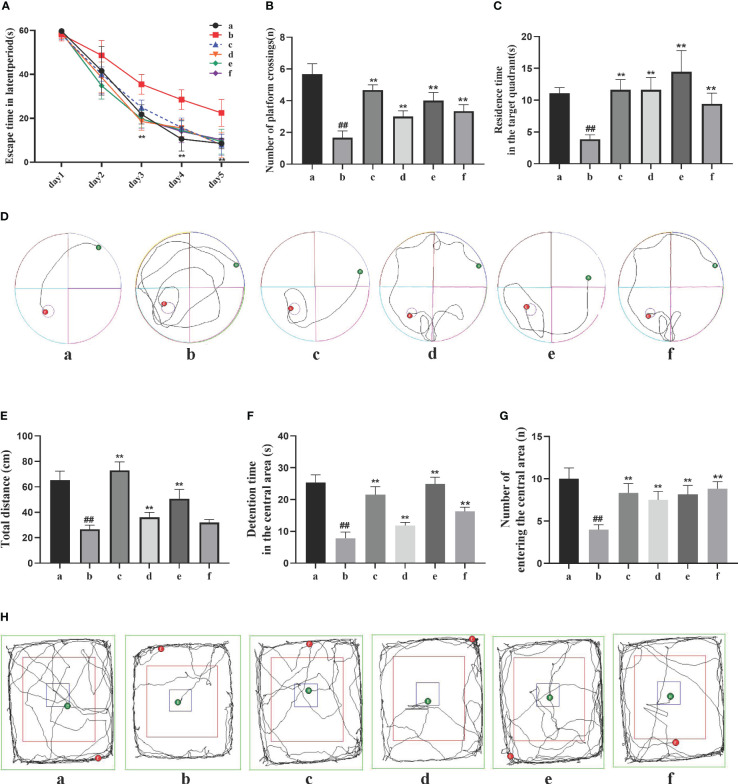
Effects of Danggui Shaoyao San and its decomposed recipes on behavior in AD rats. **(A)** Escape time in the latent period of the Morris water maze; **(B)** Number of crossings on the hidden platform area of the Morris water maze; **(C)** Stay time of the target quadrant in the Morris water maze; **(D)** Trajectories of the rats in the Morris water maze; **(E)** Total travel distance of rats in the open field test; **(F)** Time spent in the central area of the open field test; **(G)** Number of times the rat entered the central region of the open field test; **(H)** Trajectories of the rats in the open field test. The groups are represented as follows: a: sham-operated group (Sham), b: STZ injected group (AD model), c: STZ-injected DSS group (AD-DSS), and 3 DSS decomposed recipes groups: d: STZ-injected DSC group (AD-DSC), e: STZ-injected FBZ group (AD-FBZ), f: STZ-injected SB group (AD-SB). N=6 animals per group. Statistical analysis revealed significant differences compared to the sham operation group (^##^ *P* < 0.01) and the model group (**P < 0.01).

The Morris water maze test recorded the movement trajectories of AD rats during the learning phase of the first 5 days (**Figures 1A–D**). On the first day, the latency period of rats in each group was consistent, but as the experiment progressed, the average latency period showed a general decrease, with greater reduction observed in the DSS and its decomposed recipes groups compared to the model group. Compared to the sham-operated group, the model group exhibited a significant increase in latency period (*P*<0.01). Compared to the model group, the DSS, DSC, FBZ, and SB decoction groups showed a significant decrease in latency period (*P*<0.05). On the 6th day, after removing the platform, the movement trajectories of AD rats were recorded. Compared to the sham-operated group, the model group showed a reduction in the number of platform crossings and decreased time spent in the first quadrant (*P*<0.01). Compared to the model group, the DSS, DSC, FBZ, and SB decoction groups exhibited a significant increase in the number of platform crossings and a prolonged time spent in the first quadrant (*P*<0.01). Notably, the DSC group had the highest number of platform crossings, while the FBZ group had the longest time spent in the first quadrant, and these differences were statistically significant.

The open field test results (**Figures 1E–H**) showed that compared to the sham-operated group, the model group had a significantly shorter duration of activity in the central zone, reduced total distance moved, and decreased entries into the central zone (*P*<0.05). Compared to the model group, the DSS, DSC, FBZ, and SB decoction groups exhibited increased activity duration in the central zone, increased total distance moved, and increased entries into the central zone (*P*<0.05), and these differences were statistically significant. Overall, the cognitive function of AD rats was improved to some extent after oral administration treatment, and different DSS decomposed recipes groups showed varying degrees of improvement in cognitive function.

#### 3.1.2 Effects of Danggui Shaoyao San and its decomposed recipes on hippocampal neuronal function in AD rats

**Figure 2:**
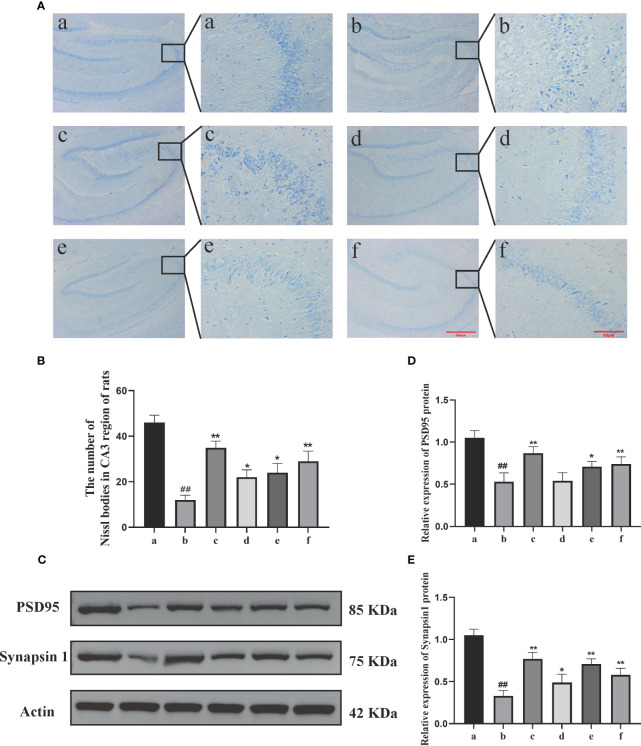
Effects of Danggui Shaoyao Powder and its decomposed recipes on hippocampal neuron function in AD rats. **(A)** Nissl’s staining; **(B)** Number of Nissl bodies in the CA3 region of rats; **(C)** Electrophoretic diagram of Western blotting; **(D)** Relative expression of synapsin I in rat hippocampus; **(E)** Relative expression of PSD95 in rat hippocampus. The groups are represented as follows: a: sham-operated group (Sham), b: STZ injected group (AD model), c: STZ-injected DSS group (AD-DSS), and 3 DSS decomposed recipes groups: d: STZ-injected DSC group (AD-DSC), e: STZ-injected FBZ group (AD-FBZ), f: STZ-injected SB group (AD-SB). N=3 animals per group. Statistical analysis revealed significant differences compared to the sham operation group (^##^ *P* < 0.01) and the model group (*P < 0.05 and **P < 0.01).

The Nissl staining results (**Figures 2A, B**) showed that in the sham-operated group, the neuronal cells exhibited normal size and quantity, regular morphology, and a compact and orderly arrangement. The cytoplasm showed evenly stained Nissl bodies. In the model group, the neuronal cells displayed vacuolar changes or shrinkage, a significant reduction in quantity, decreased Nissl bodies, lighter staining, a loose arrangement, and nuclear dissolution. In the DSS, DSC, FBZ, and SB groups, the neuronal arrangement was relatively regular. Compared to the model group, the DSS, FBZ, and SB groups showed an increase in the number of Nissl bodies in neurons, reduced cell swelling or shrinkage, and an increase in intracellular Nissl bodies (*P*<0.05).

Western blot analysis was performed to detect the expression of synaptic proteins PSD95 and synapsin I in the hippocampal region of different groups (**Figure 2C**). Compared to the sham-operated group, the model group exhibited a decrease in the expression levels of synaptic proteins PSD95 (*P*<0.01) and synapsin I (*P*<0.05) (**Figures 2D, E**). Compared to the model group, the DSS, DSC, FBZ, and SB groups showed an increase in synapsin I expression (*P*<0.01, *P*<0.05) and an increase in PSD95 expression (*P*<0.01, *P*<0.05) (**Figures 2D, E**). Overall, the expression levels of synapsin I and PSD95 increased in different DSS decomposed recipes groups, but the extent of increase varied among the groups, and these differences were statistically significant.

### 3.2 Effect of Danggui-Shaoyao-San on gut microbiota

#### 3.2.1 Alpha diversity and beta diversity analysis

**Figure 3:**
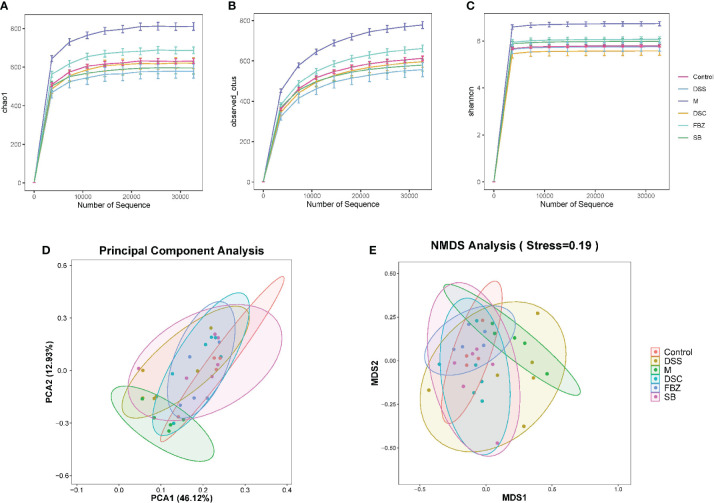
Effects of Danggui Shaoyao San and its decomposed recipes abundance and diversity of intestinal flora in AD rats. Alpha diversity analysis of intestinal flora richness and evenness. The Chao1 **(A)** and Observed_otus **(B)** indexes estimate the number of species contained in the intestinal flora. The Shannon **(C)** index estimates microbial diversity in the intestinal flora. Beta diversity analysis of intestinal flora of different species. **(D)** Principal component analysis (PCA); **(E)** Non-metric multidimensional scaling analysis (NMDS). The groups are represented as follows: sham-operated group (Control), STZ injected group (M), STZ-injected DSS group (DSS), and 3 DSS decomposed recipes groups: STZ-injected DSC group (DSC), STZ-injected FBZ group (FBZ), STZ-injected SB group (SB).

Alpha diversity analysis primarily reflects the species abundance and diversity of gut microbiota. The Chao1 and Observed_otus indices reflect the species abundance in the samples. The results (**Figures 3A, B**) showed that the FBZ group had the richest diversity of gut microbiota among the groups after oral administration. The Shannon index reflects species diversity. A higher Shannon value indicates higher species diversity. The results (**Figure 3C**) demonstrated that the gut microbiota of AD rats was disrupted, and species diversity was disrupted. After oral administration, the FBZ group exhibited the highest species diversity. This suggests that after oral administration in AD rats, the gut microbiota in the FBZ group was closer to that of the sham-operated group in terms of species diversity.

Beta diversity analysis mainly reflects the species differences in gut microbiota among the groups. The results (**Figures 3D, E**) showed significant differences between the model group and the sham-operated group, indicating different microbial compositions. After medication intervention, the differences between each group and the sham-operated group decreased, with the DSC group being the closest to the sham-operated group and showing the smallest difference. This indicates that DSS and its decoctions caused significant changes in the abundance and composition of gut microbiota in AD rats, and different decoction groups had different effects on regulating the composition of gut microbiota.

#### 3.2.2 Differential species analysis

**Figure 4:**
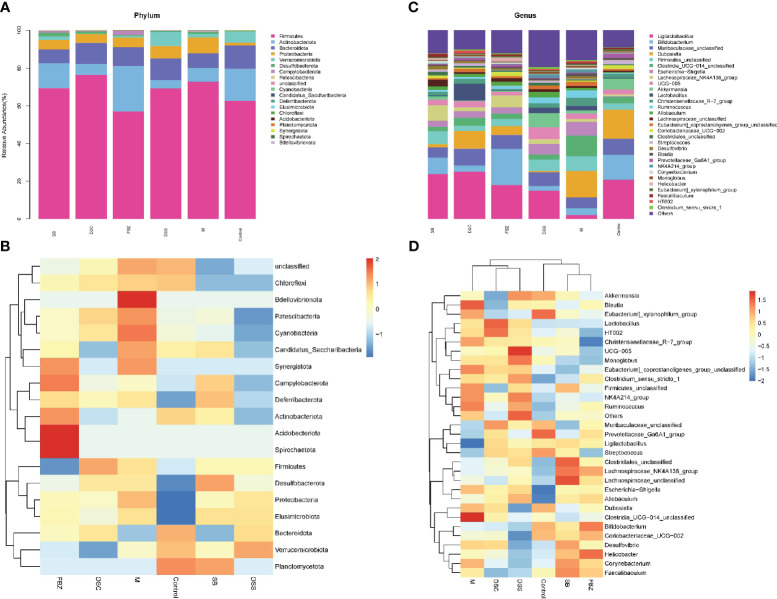
Species analysis of intestinal flora. **(A)** Columnar stack diagram of species composition at the Phylum level; **(B)** Columnar stack diagram of species composition at the genus level; **(C)** Heat map of species composition at the Phylum level; **(D)** Heat map of species composition at the genus level. The groups are represented as follows: sham-operated group (Control), STZ injected group (M), STZ-injected DSS group (DSS), and 3 DSS decomposed recipes groups: STZ-injected DSC group (DSC), STZ-injected FBZ group (FBZ), STZ-injected SB group (SB).

The analysis of samples at the phylum level showed (**Figures 4A, B**, **Supplementary Table 1**) that the sham-operated group had higher abundance of the phyla Firmicutes, Bacteroidota, and Actinobacteriota, and lower abundance of Proteobacteria and Verrucomicrobia, while the remaining phyla had very low abundance. Compared to the sham-operated group, the model group exhibited an increase in the abundance of Firmicutes and Proteobacteria in the gut microbiota, and a decrease in Actinobacteriota, Bacteroidota, and Verrucomicrobia. After medication treatment, the structure of gut microbiota in AD rats at the phylum level underwent changes. Compared to the model group, the Bacteroides increased the most in the DSS group, Firmicutes increased the most in the DSC group, and Verrucomicrobia increased the most in the FBZ group. Overall, the types of phyla in the gut microbiota remained relatively unchanged after medication intervention in rats, but the abundance showed significant changes, with different DSS decomposed recipes groups affecting different phyla.

The analysis of samples at the genus level showed (**Figures 4C, D**, **Supplementary Table 2**) that the sham-operated group had higher abundance of genera such as Ligilactobacillus, Bifidobacterium, Dubosiella, Muribaculaceae, and Akkermansia. Compared to the sham-operated group, the model group exhibited increased abundance of Firmicutes, Escherichia-Shigella, Corynebacterium, Lactobacillus, Desulfovibrio, Blautia, and Helicobacter at the genus level, while genera such as Lactobacillus, Bifidobacterium, Akkermansia, and Ducella decreased. Compared to the model group, the gut microbiota underwent changes after oral administration, with the DSC group showing the highest increase in Lactobacillus_reuteri, the FBZ group showing the highest increase in Bifidobacteria, the DSS group showing thehighest increase in Akkermansia, the SB group showing the highest increase in Trichobacterium, and the DSS group showing the largest decrease in Helicobacter_pylori. Various groups also showed varying degrees of decrease in Lactic acid bacteria, Escherichia-Shigella, and Rumen species. In summary, the gut microbiota at the genus level in rats underwent significant changes in composition and abundance after intervention with DSS and its decomposed recipes.

#### 3.2.3 Biomarker screening

**Figure 5:**
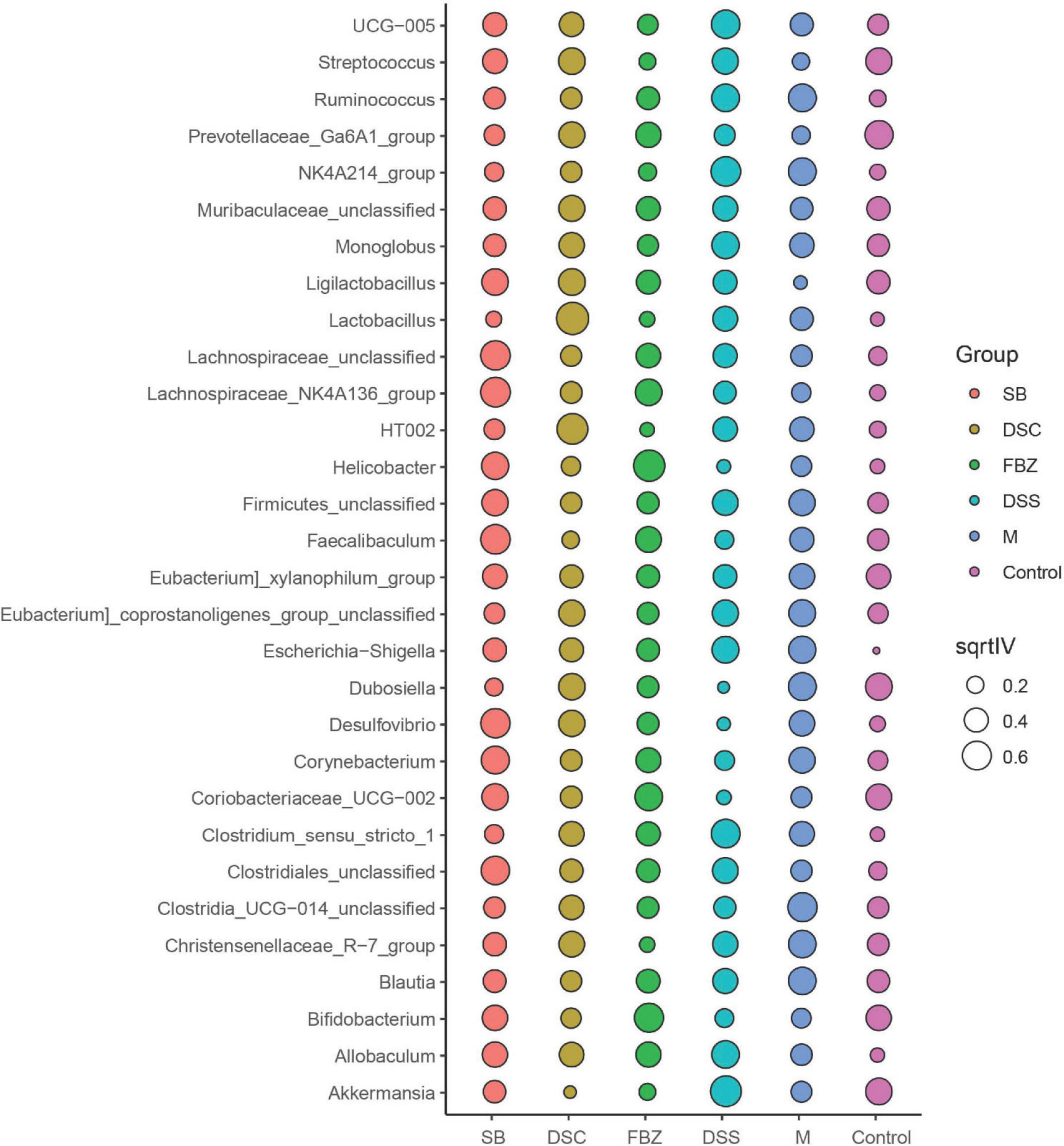
Biomarkers in the top 30 species were determined by indicator analysis. Streptococcus, Ruminococcus, Ga6A1, NK4A214, Ligilactobacillus, Lactobacillus, Escherichia-Shigella, Clostridium, and Allobaculum are potential biomarkers for AD. The groups are represented as follows: sham-operated group (Control), STZ injected group (M), STZ-injected DSS group (DSS), and 3 DSS decomposed recipes groups: STZ-injected DSC group (DSC), STZ-injected FBZ group (FBZ), STZ-injected SB group (SB).

To identify potential biomarkers, analysis was performed using the top 30 abundant species at genus level. The results (**Figure 5**, **Supplementary Table 3**) showed that compared to the sham-operated group, species such as Streptococcus, Ruminococcus, Ga6A1, NK4A214, Ligilactobacillus, Lactobacillus, Escherichia-Shigella, Clostridium, and Allobaculum could serve as potential biomarkers for AD. DSS and its decomposed recipes were able to improve the abundance of these microbial species. For example, the DSS group and SB group significantly improved the abundance of Allobaculum. The FBZ group and SB group significantly improved the abundance of Lactobacillus. All groups significantly improved the abundance of Escherichia-Shigella, with the FBZ group showing the most pronounced effect. The DSS group, DSC group, and SB group significantly improved the abundance of Streptococcus. The FBZ group, DSC group, and SB group significantly improved the abundance of Ruminococcus.

#### 3.2.4 Correlation analysis

**Figure 6:**
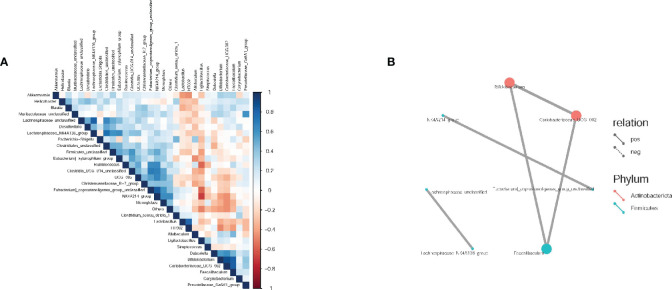
Spearman correlation analysis of intestinal flora in rats. **(A)** Correlation heat maps between dominant flora at the genus level; **(B)** Correlation network diagram between dominant flora at the genus level. The groups are represented as follows: sham-operated group (Control), STZ injected group (M), STZ-injected DSS group (DSS), and 3 DSS decomposed recipes groups: STZ-injected DSC group (DSC), STZ-injected FBZ group (FBZ), STZ-injected SB group (SB).

Spearman correlation analysis was conducted to evaluate the correlation and significance (*P*-values) between the top 30 dominant genera at the genus level. As shown in **Figure 6**, a positive correlation was observed between Bifidobacterium, Erythromycetes - UCG-002 (both belonging to Actinomycetes), and Pseudobacterium. Additionally, a positive correlation was found between Rumen species NK4A214 group and Pecalsterol-producing eurod bacteria.

#### 3.2.5 BugBase bacterial phenotype prediction

**Figure 7:**
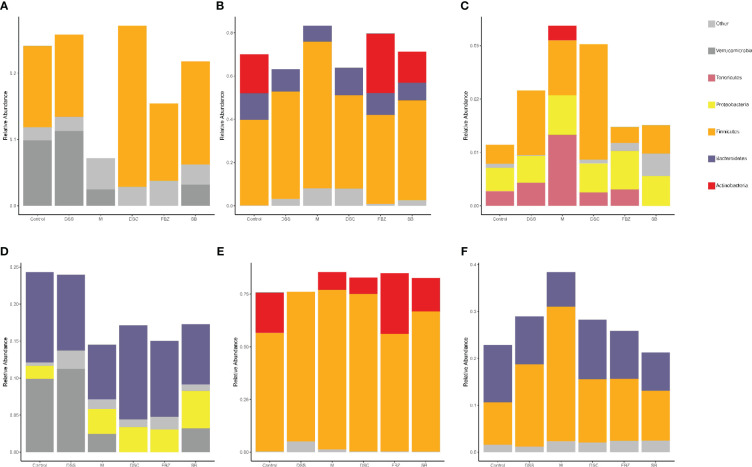
Bacterial phenotype prediction. **(A)** Aerobic phenotype; **(B)** Anaerobic phenotype; **(C)** Facultatively Anaerobic phenotype; **(D)** Gram Negative phenotype; **(E)** Gram positive phenotype; **(F)** potentially pathogenic phenotype. The groups are represented as follows: sham-operated group (Control), STZ injected group (M), STZ-injected DSS group (DSS), and 3 DSS decomposed recipes groups: STZ-injected DSC group (DSC), STZ-injected FBZ group (FBZ), STZ-injected SB group (SB).

BugBase analysis was conducted to predict the phenotypes of the microbiome samples, including 9 potential phenotypes. In this study, 6 potential phenotypes were selected for investigation, including Aerobic, Anaerobic, Facultatively Anaerobic, Gram Negative, Gram Positive, and Potentially Pathogenic. As shown in **Figure 7** and **Supplementary Table 4**, in the Aerobic category, the DSC group had the highest relative abundance, while the model group had a significantly lower relative abundance compared to other groups. In the Anaerobic category, there was no significant difference in relative abundance among the groups, except that Actinomycetes were absent in the model group, DSC group, and DSS group. In the Facultatively Anaerobic category, the model group had a significantly higher relative abundance, and Actinomycetes were present, while the other groups did not have Actinomycetes. In the Gram Negative category, the model group had the lowest relative abundance, and the DSS group did not have Proteobacteria, whereas the other groups had Proteobacteria. In the Gram Positive category, the relative abundances were similar among the groups. In the Potentially Pathogenic category, the model group had the highest relative abundance, with a significant increase in Firmicutes, indicating a higher abundance of potentially pathogenic bacteria in the model group, making it more susceptible to disease.

## 4 Discussion

Alzheimer’s disease (AD) is classified in Traditional Chinese Medicine as “shānwàng” (memory impairment), “dāibìng” (cognitive dysfunction), and “chīdāi” (dementia). The pathogenesis of AD is multifactorial and not well understood. The mainstream hypothesis, the amyloid-β (Aβ) hypothesis, has led to the development of drugs that have only reached clinical trials without success (Sun et al., 2018; Murayama, 2023). The gut microbiota plays a role in the pathogenesis of AD and is involved in the cognitive and AD-related mechanisms in the host. Dysbiosis of the gut microbiota can lead to increased intestinal and blood-brain barrier permeability, which may mediate or influence the pathogenesis of AD and other neurodegenerative diseases, particularly those related to aging. Furthermore, bacteria in the gut microbiota can secrete abundant amyloid-like proteins and lipopolysaccharides, which may contribute to the regulation of signaling pathways and the production of pro-inflammatory cytokines associated with AD pathogenesis (Angelucci et al., 2019). Therefore, research on the gut microbiota has become a new direction in the prevention and treatment of AD.

Cognitive dysfunction is the primary clinical symptom of Alzheimer’s disease, but recent research has shown that Alzheimer’s patients experience other neuropsychiatric symptoms (NPS) alongside cognitive impairment, such as anxiety and depression (Murayama et al., 2021). These additional symptoms are attributed to overlapping mechanisms shared by neurodegenerative diseases (NDD) and neuropsychiatric disorders (NPD), including post-translational modifications, the microbiota-gut-brain axis, and signaling events (Gupta et al., 2023). In this study, the Morris water maze (MWM) test revealed that the AD rat model induced by the lateral ventricle injection of STZ displayed sensitivity to anxiety and depression, and this anxiety and depression-like behavior was improved by DSS. Previous studies have suggested that DSS promotes mitophagy and inhibits the inflammatory activation of microglia as potential mechanisms for improving depression-like behavior (Song et al., 2021b; Yang et al., 2023). The significance of changes in the gut microbiota and their impact on the course of neurological diseases has gained increased attention. Studies have demonstrated that the microbiota-gut-brain axis is essential for normal nervous system function (Zhou et al., 2023). Interventions involving prebiotics, probiotics, or Biostime have shown that the microbiota plays a crucial role in neurological diseases by synthesizing neuroactive compounds that interact with the nervous system and regulating inflammatory and endocrine processes (Borrego-Ruiz and Borrego, 2024). Therefore, the regulation of intestinal flora by DSS may be an important mechanism to improve cognitive dysfunction in Alzheimer’s disease.

Studies have shown significant differences in the composition of the gut microbiota between AD patients and normal control groups. In clinical settings, AD patients have been found to have increased levels of Bacteroides and decreased levels of Firmicutes (Vogt et al., 2017; Sochocka et al., 2019). The gut-brain axis, which involves the autonomous regulation of gastrointestinal physiology and function through the vagus nerve pathway, plays a bidirectional communication role with the central nervous system, forming a “gut-brain axis” (Kesika et al., 2021). Research has indicated that gut lactobacilli can exert neuroprotective effects by influencing neurotransmitter release in neurons or affecting blood lipid levels (Wu et al., 2018). Certain bacteria, such as those that produce short-chain fatty acids (SCFAs), have been found to have a positive correlation between their levels and cognitive function (Silva et al., 2020). Probiotics can alleviate cognitive impairment in AD by inhibiting the synthesis of trimethylamine-N-oxide, downregulating Aβ levels in the hippocampus, and protecting neurons from damage (Wang et al., 2020). Helicobacter pylori, which can enter the brain via the oral-nasal-olfactory pathway, has been implicated in neurodegeneration and can influence the course of Alzheimer’s disease (Roubaud Baudron et al., 2016). The results of this study demonstrated significant changes in the abundance and composition of the gut microbiota in AD rats following treatment with DSS and its decomposed recipes. The composition, abundance, and diversity of the gut microbiota in the DSS group and its decomposed recipes groups were similar to those in the sham-operated group. The Akkermansia genus showed the highest increase, while Helicobacter pylori showed the greatest decrease. The groups receiving different individual components had varying degrees of regulation on the composition of the gut microbiota, with the Lactobacilli genus showing the highest increase in the DSC group, Bifidobacteria showing the highest increase in the FBZ group, and Trichobacterium showing the highest increase in the SB group. These findings suggest that DSS and its decomposed recipes regulate the gut microbiota by reducing the abundance of harmful bacteria such as Bacteroides and Helicobacter pylori and increasing the abundance of beneficial bacteria such as Akkermansia, Lactobacilli, and Trichobacterium, thereby improving cognitive learning and reducing anxiety-like behavior in AD rats.

The composition and abundance of the gut microbiota refer to the components and quantities of microbial communities in the human gut. The composition and abundance of the gut microbiota can influence various physiological functions of the human body, includingdigestion, immunity, and metabolism, and are closely associated with the development of many diseases (Cryan et al., 2019). Therefore, specific changes in the gut microbiota can serve as a way to explore disease-related biomarkers and may be used to identify co-varying microbial dysbiosis patterns driving the occurrence of various diseases. Most biomarkers are not causative factors of a single disease but are associated with multiple diseases, and microbial dysbiosis patterns may provide important clues for the occurrence of different diseases (Chen et al., 2021). This study suggests that Akkermansia, Lactobacillus, Escherichia-Shigella, and Rumen species may serve as potential biomarkers for AD. The prediction of bacterial phenotypes revealed that potential pathogenic bacteria had the highest relative abundance in AD rats, and Firmicutes showed a significant increase, indicating a close relationship between the changes in the abundance of certain pathogenic bacteria in the gut and the progression of AD. Furthermore, significant changes in the abundance and composition of the gut microbiota were observed in AD rats following intervention with DSS and its decomposed recipes. Among them, the SB group had the lowest relative abundance of potential pathogenic bacteria.

The study presented important findings related to the potential use of TCM in the treatment of AD by focusing on the impact of DSS and its decomposed recipes on cognitive function and the gut microbiota of AD rats. Gut microbiota composition and abundance changes following treatment with DSS and its decomposed recipes suggest that specific bacteria, such as Akkermansia, Lactobacillus, and Trichobacterium, are associated with improved cognitive function. This finding opens up opportunities for future investigations on probiotics and prebiotics in the context of AD treatment. Moreover, the identification of potential biomarkers for AD in the gut microbiota, including Akkermansia, Lactobacillus, Escherichia-Shigella, and Rumen species, is of great significance. This finding could be applied in clinical practice for early diagnosis and monitoring of AD by examining the gut microbiota composition in patients. In clinical practice, our results may inform the development of TCM-based treatments for AD, focusing on the use of DSS or its components. The compatibility of TCM prescriptions and the selection of specific herbs or compounds to regulate gut microbiota and improve cognitive function could lead to innovative approaches in AD management.

## 5 Conclusions

In conclusion, our study reveals significant alterations in the gut microbiota of Alzheimer’s disease (AD) rats, concurrent with observed neuronal damage in the brain and impaired cognitive function. Furthermore, we have demonstrated that DSS and its decomposed recipes have the potential to modulate the gut microbiota, favoring beneficial bacterial genera such as Akkermansia, Lacticacidbacteria, and Trichobacterium, while reducing the abundance of detrimental bacterial genera such as Bacteroides and Helicobacter pylori. These alterations in the gut microbiota are associated with a reduction in neuronal damage within the hippocampus and an improvement in cognitive function among AD rats.

Our findings not only shed light on the differential therapeutic effects of DSS and its decomposed recipes but also offer insights into their potential role in the field of Alzheimer’s treatment. The observed microbiota changes suggest that traditional Chinese medicine, specifically DSS and its decomposed recipes, may hold promise as a complementary approach to AD therapy. Moreover, our study paves the way for future research, as it underscores the need to delve deeper into the precise mechanisms by which the whole prescription (DSS) and its decomposed recipes exert their influence on the gut microbiota. Further investigations are warranted to identify the specific components responsible for these effects, which will enhance our understanding and guide clinical prescription compatibility more effectively.

## Data availability statement

The datasets presented in this study can be found in online repositories. The names of the repository/repositories and accession number(s) can be found below: https://www.ncbi.nlm.nih.gov/, PRJNA1029816.

## Ethics statement

The animal study was approved by the Ethics Committee of Hunan University of Chinese Medicine. The study was conducted in accordance with the local legislation and institutional requirements.

## Author contributions

YJ: Conceptualization, Data curation, Formal Analysis, Methodology, Writing – original draft, Writing – review & editing. SL: Data curation, Investigation, Methodology, Writing – original draft. JQ: Data curation, Formal Analysis, Investigation, Methodology, Writing – original draft. JJ: Data curation, Formal Analysis, Methodology, Writing – original draft. YZ: Data curation, Formal Analysis, Methodology, Writing – original draft. YH: Data curation, Investigation, Methodology, Writing – original draft. CH: Data curation, Investigation, Methodology, Writing – review & editing. WY: Data curation, Formal Analysis, Investigation, Writing – review & editing. SD: Data curation, Investigation, Methodology, Writing – original draft. SC: Resources, Supervision, Writing – review & editing, Funding acquisition, Project administration, Validation. ZS: Funding acquisition, Project administration, Resources, Supervision, Validation, Writing – review & editing, Conceptualization.

## Supplementary material

Supplementary Figure 1 The original image of western blotting.
