# Supplementary figures and images for: Intestinal flora study reveals the mechanism of Danggui Shaoyao San and its decomposed recipes to improve cognitive dysfunction in the rat model of Alzheimer’s disease
Source: Front Cell Infect Microbiol. 2023 Nov 21;13:1323674. doi: 10.3389/fcimb.2023.1323674 (PMC10699443; doi:10.3389/fcimb.2023.1323674)

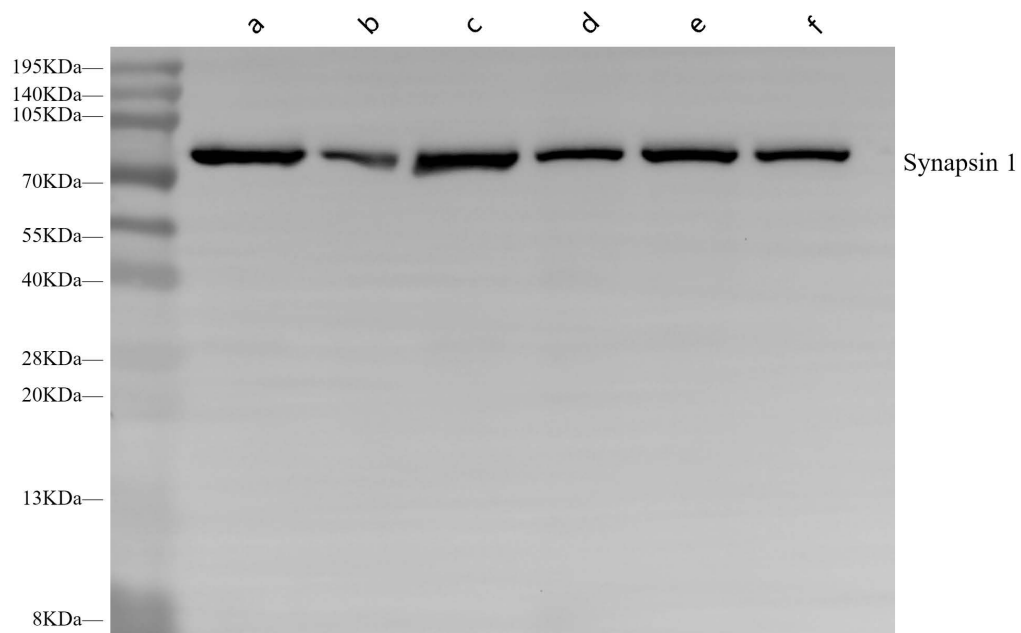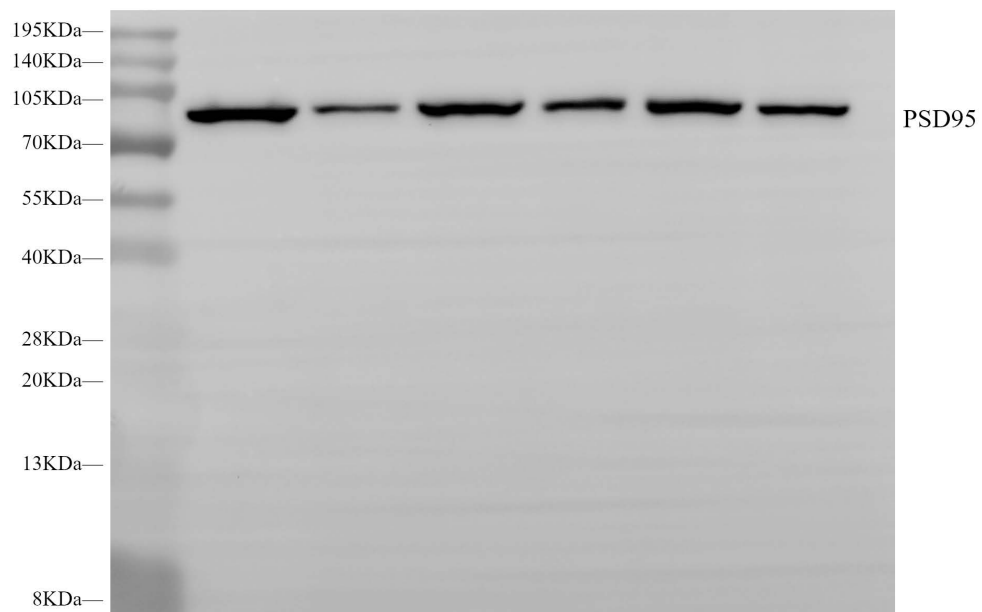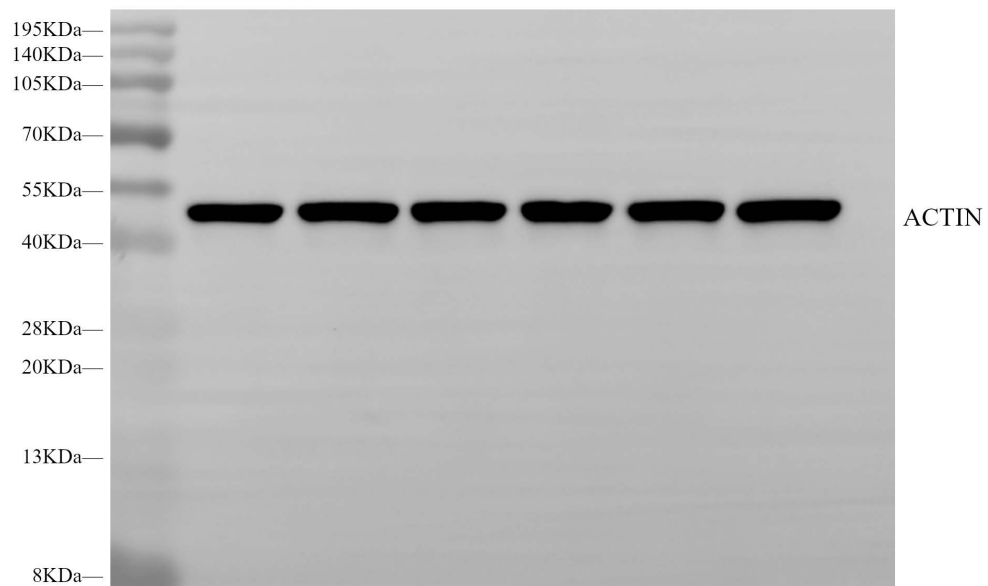

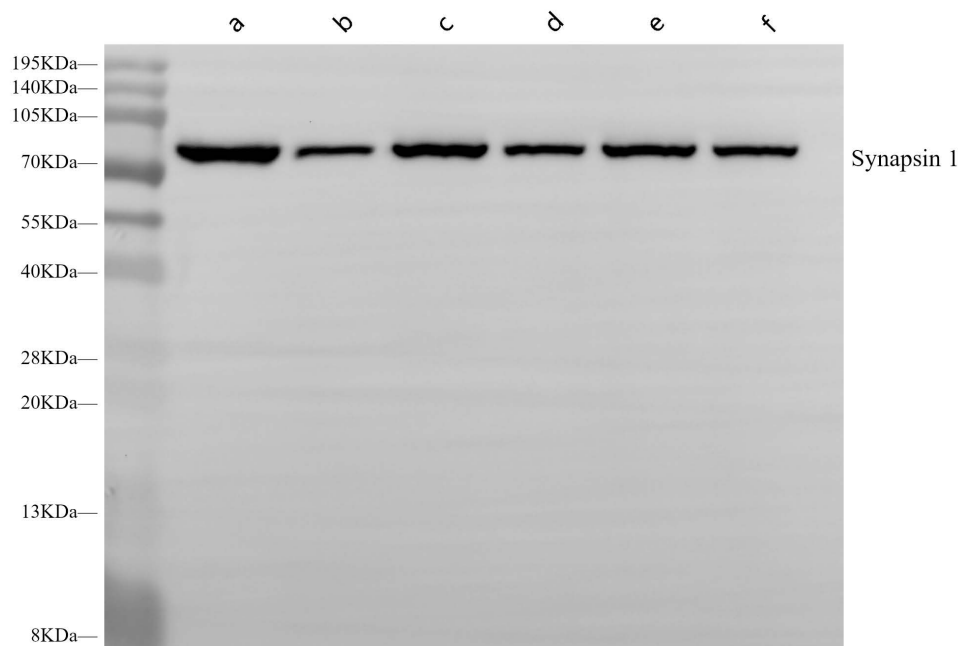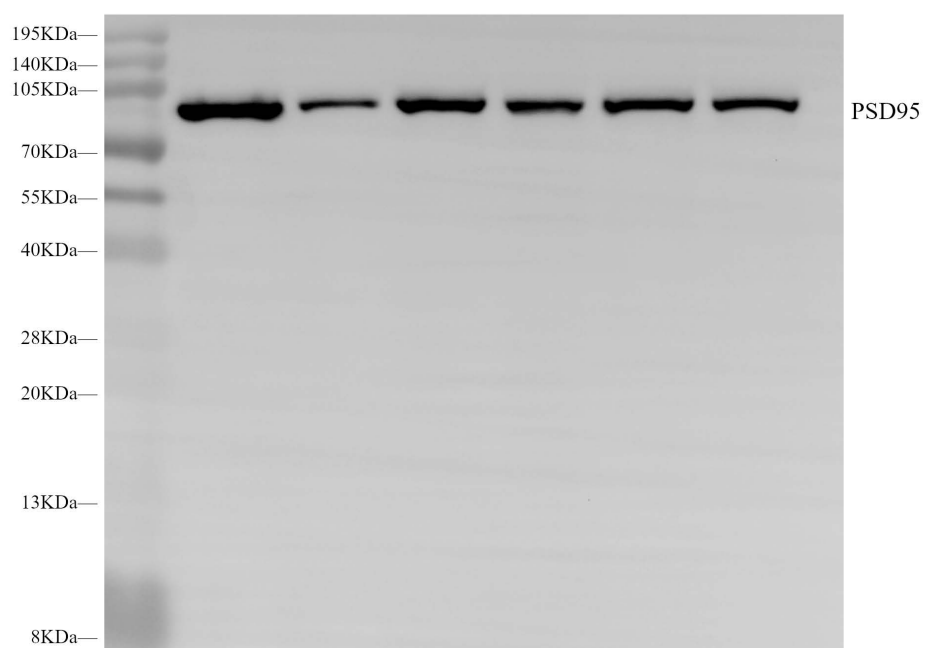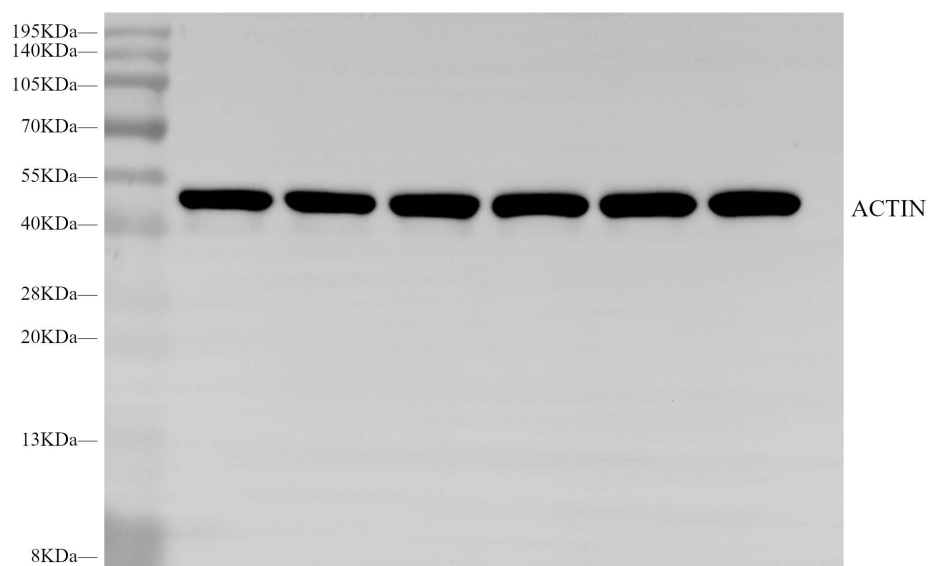

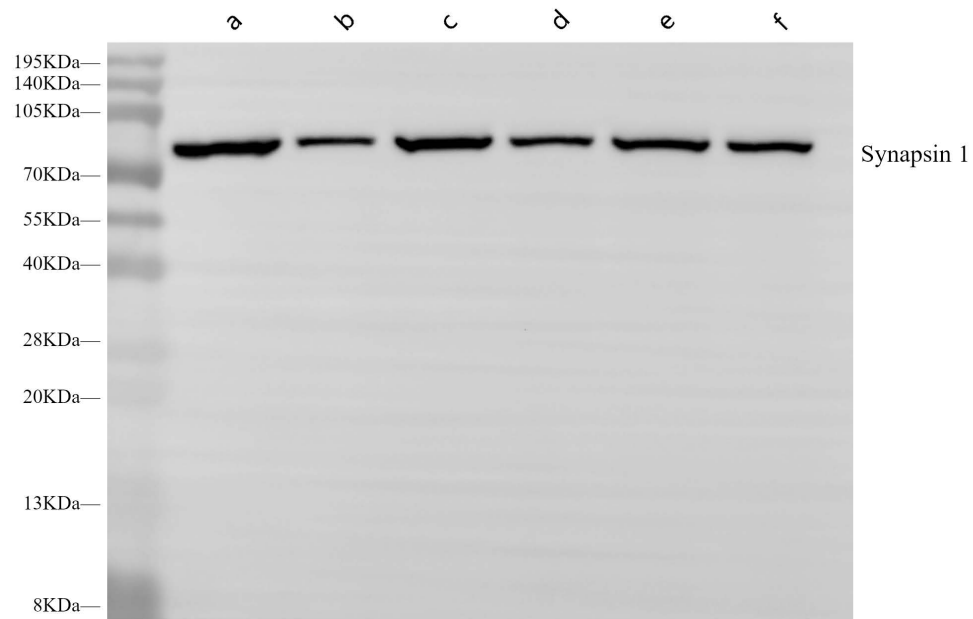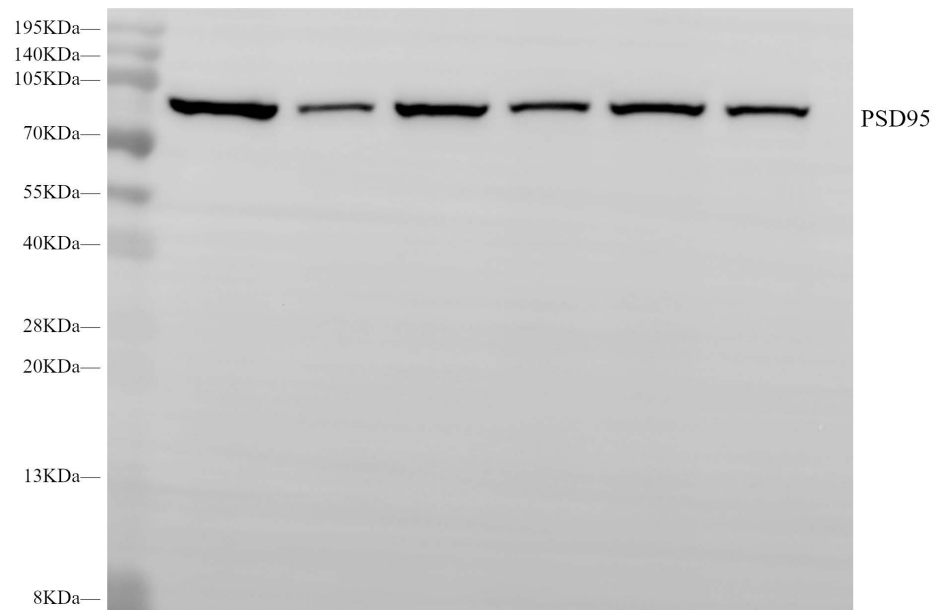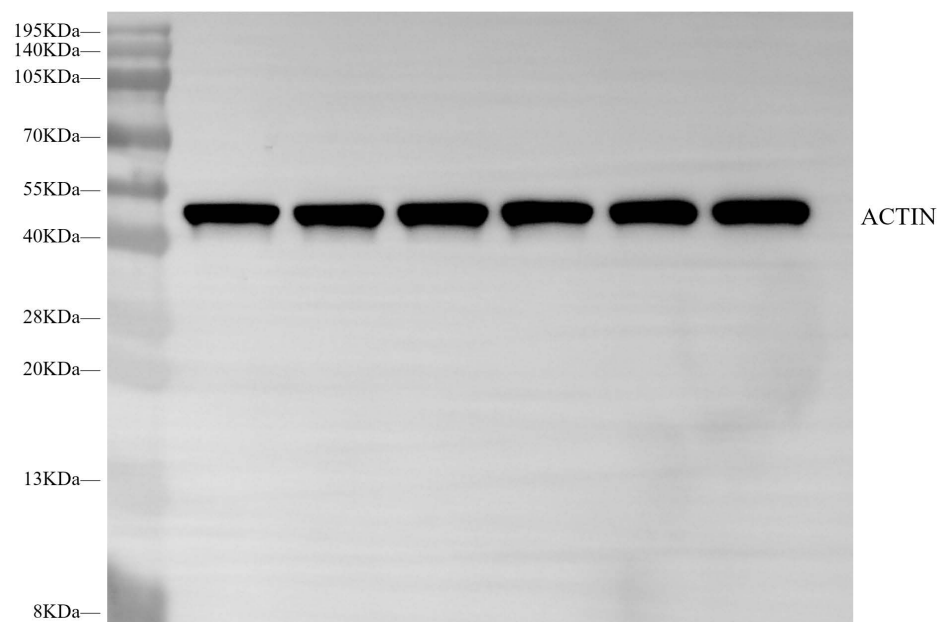

Supplement: Supplementary Figure 1 — The original image of western blotting. [file DataSheet_1.pdf]
