# Supplementary material for: Intestinal flora study reveals the mechanism of Danggui Shaoyao San and its decomposed recipes to improve cognitive dysfunction in the rat model of Alzheimer’s disease
Source: Front Cell Infect Microbiol. 2023 Nov 21;13:1323674. doi: 10.3389/fcimb.2023.1323674 (PMC10699443; doi:10.3389/fcimb.2023.1323674)
Supplement: Supplementary file 2 [file DataSheet_2.zip › statistical programs/16S rDNA project materials and methods.pdf]

Material and method description of 16SrDNA sequencing

DNA extractions

DNA from different samples was extracted using the CTAB according to manufacturer 's instructions. The reagent which was designed to uncover DNA from trace amounts of sample has been shown to be effective for the preparation of DNA of most bacteria. Nuclear-free water was used for blank. The total DNA was eluted in 50 μL of Elution buffer and storedat -80 °C until measurement in the PCR.

PCR amplification and 16S rDNA sequencing

Region	Primers
V3-V4[1]	341F (5'-CCTACGGGNGGCWGCAG-3')<br>805R(5'-GACTACHVGGGTATCTAATCC-3')
Archae[3]	F(5'-GYGCASCAGKCGMGAAW-3')<br>R(5'-GGACTACHVGGGTWTCTAAT-3')
V4[2]	515F(5'-GTGYCAGCMGCCGCGGTAA-3')<br>806R (5'- GGACTACHVGGGTWTCTAAT-3')
V4-V5	F(5'-GTGCCAGCMGCCGCGG-3')<br>R(5'-CCGTCAATTCMTTTRAGTTT-3')

The 5' ends of the primers were tagged with specific barcods per sample and sequencing universal primers.PCR amplification was performed in a total volume of 25 μL reaction mixture containing 25 ng of template DNA, 12.5 μL PCR Premix, 2.5 μL of each primer, and PCR-grade water to adjust the volume. The PCR conditions to amplify the prokaryotic 16S fragments consisted of an initial denaturation at 98 °C for 30 seconds; 32cycles of denaturation at 98 °C for 10 seconds, annealing at 54°C for 30 seconds, and extension at 72 °C for 45 seconds; and then final extension at 72 °C for 10 minutes. The PCR products were confirmed with 2% agarose gel electrophoresis. Throughout the DNA extraction process, ultrapure water, instead of a sample solution, was used to exclude the possibility of false-positive PCR results as a negative control. The PCR products were purifyied by AMPure XT beads (Beckman Coulter Genomics, Danvers, MA, USA) and quantified by Qubit(Invitrogen, USA). The amplicon pools were prepared for sequencing and the size and quantity of the amplicon library were assessed on Agilent 2100 Bioanalyzer (Agilent, USA) and with the Library Quantification Kit for Illumina (Kapa Biosciences, Woburn, MA, USA), respectively. The libraries were sequenced on NovaSeq PE250 platform.

Data analysis

Samples were sequenced on an Illumina NovaSeq platform according to the manufacturer's recommendations. Paired-end reads was assigned to samples based on their unique barcode and truncated by cutting off the barcode and primer sequence. Paired-end reads were merged using FLASH. Quality filtering on the raw reads were performed under specific

filtering conditions to obtain the high-quality clean tags according to the fqtrim(v0.94). Chimeric sequences were filtered using Vsearch software(v2.3.4). After dereplication using DADA2,we obtained feature table and feature sequence.Alpha diversity and beta diversity were calculated by normalized to the same sequences randomly.Then according to SILVA(release 138) classifier, feature abundance was normalized using relative abundance of each sample . Alpha diversity is applied in analyzing complexity of species diversity for a sample through 5 indices, including Chao1, Observed species, Goods coverage, Shannon, Simpson, and all this indices in our samples were calculated with QIIME2. Beta diversity were calculated by QIIME2,the graphs were drew by R package.Blast was used for sequence alignment, and the feature sequences were annotated with SILVA database for each representative sequence.Other diagrams were implemented using the R package(v3.5.2).

Reference：

[1] Logue Jürg B,Stedmon Colin A,Kellerman Anne M et al. Experimental insights into the importance of aquatic bacterial community composition to the degradation of dissolved organic matter.[J] .ISME J, 2016, 10: 533-45.
[2] Walters W, Hyde E R, Berglyons D, et al. Improved Bacterial 16S rRNA Gene (V4 and V4-5) and Fungal Internal Transcribed Spacer Marker Gene Primers for Microbial Community Surveys:[J]. Msystems, 2015, 1(1):e00009-15.
[3] KEN TAKAI* AND KOKI HORIKOSHI.Rapid Detection and Quantification of Members of the Archaeal Community by Quantitative PCR Using Fluorogenic Probes. APPLIED AND ENVIRONMENTAL MICROBIOLOGY Nov. 2000, p. 5066–5072.
